# Analytical workflow of double-digest restriction site-associated DNA sequencing based on empirical and *in silico* optimization in tomato

**DOI:** 10.1093/dnares/dsw004

**Published:** 2016-02-29

**Authors:** Kenta Shirasawa, Hideki Hirakawa, Sachiko Isobe

**Affiliations:** Kazusa DNA Research Institute, 2-6-7 Kazusa-Kamatari, Kisarazu, Chiba292-0818, Japan

**Keywords:** genetic linkage map, restriction-associated DNA sequencing, single nucleotide polymorphism, tomato (*Solanum lycopersicum*), *in silico* prediction

## Abstract

Double-digest restriction site-associated DNA sequencing (ddRAD-Seq) enables high-throughput genome-wide genotyping with next-generation sequencing technology. Consequently, this method has become popular in plant genetics and breeding. Although computational *in silico* prediction of restriction sites from the genome sequence is recognized as an effective approach for choosing the restriction enzymes to be used, few reports have evaluated the *in silico* predictions in actual experimental data. In this study, we designed and demonstrated a workflow for *in silico* and empirical ddRAD-Seq analysis in tomato, as follows: (i) *in silico* prediction of optimum restriction enzymes from the reference genome, (ii) verification of the prediction by actual ddRAD-Seq data of four restriction enzyme combinations, (iii) establishment of a computational data processing pipeline for high-confidence single nucleotide polymorphism (SNP) calling, and (iv) validation of SNP accuracy by construction of genetic linkage maps. The quality of SNPs based on *de novo* assembly reference of the ddRAD-Seq reads was comparable with that of SNPs obtained using the published reference genome of tomato. Comparisons of SNP calls in diverse tomato lines revealed that SNP density in the genome influenced the detectability of SNPs by ddRAD-Seq. *In silico* prediction prior to actual analysis contributed to optimization of the experimental conditions for ddRAD-Seq, e.g. choices of enzymes and plant materials. Following optimization, this ddRAD-Seq pipeline could help accelerate genetics, genomics, and molecular breeding in both model and non-model plants, including crops.

## 1. Introduction

DNA markers are essential tools for molecular genetics and genomics. Simple sequence repeats (SSRs, also called microsatellites) and single nucleotide polymorphisms (SNPs) are the most powerful and widely used DNA markers. SSRs have the advantages of being both co-dominant and multi-allelic in nature, but they require time-consuming gel or capillary electrophoresis analyses. On the other hand, SNPs, most of which are co-dominant but bi-allelic, can be analysed using time-saving gel-free techniques, e.g. TaqMan assays,^1^ Kompetitive Allele-Specific PCR (KASP; LGC, London, UK), and high-resolution melting analysis (Idaho Technology, Salt Lake City, UT). Microarray-based SNP chip technologies, e.g. GoldenGate and Infinium (Illumina, San Diego, CA), and Axiom (Affymetrix, Santa Clara, CA), have enabled high-throughput SNP genotyping and thereby contributed to statistical genetic approaches, e.g. quantitative trait locus analyses and genome-wide association studies.^2,3^ However, SNP microarrays have a disadvantage, namely, the lack of flexibility in experimental design. Progress in next-generation sequencing (NGS) technology has enabled the development of huge numbers of SNPs in both model and non-model plant species, including crops.^4^ Correspondingly, SNP genotyping by NGS, e.g. genotyping by sequencing (GBS) and restriction site-associated DNA sequencing (RAD-Seq), have recently become popular due to their flexibility and relatively low cost.^5^

GBS was initially developed in maize^6^ and subsequently applied to other crop species.^7^ In the original GBS protocol, genomic DNA is digested with restriction enzymes, and adapters are ligated to the restriction ends.^6^ Sequencing data of single-end reads are always obtained from sites associated with the restriction ends, which is a great advantage in sequencing of identical loci across multiple samples. The RAD-Seq method,^8^ which is similar to GBS, has been applied to several plant species.^5,7^ In the original RAD-Seq protocol, genomic DNA is fragmented twice by different methods: first by a restriction enzyme, and second by physical shearing. The resultant DNA fragments, with restriction sites on one end and sheared ends on the other, are targeted for single-end sequencing analysis from the restriction ends. On the other hand, paired-end sequence reads can be more accurately mapped onto the reference genome than single-end reads, especially in plants, which often have large and complex polyploid genomes.^9^ Double-digest restriction site-associated DNA-Seq (ddRAD-Seq), in which a second restriction enzyme is employed for digestion of genome DNA to reduce cost and time to prepare the sequencing libraries, enables paired-end sequencing of identical loci across multiple samples.^10^ Therefore, from the point of view of high accuracy read mapping even in the complex plant genomes, ddRAD-Seq technology has the advantage over GBS and RAD-Seq. Along with the great advances in the sequencing technology, several data processing pipelines for GBS and RAD-Seq have been reported.^11,12^ However, as mentioned, plants have complex genomes due to many types of ploidy, reproduction systems as well as various genome sizes. Therefore, data processing methods with flexibility in manipulation would be required.

Whole-genome sequencing (WGS) analysis of several plant species has been accelerated by NGS technology; as of June 2015, genome sequence data are available from >100 plants.^13^ This situation makes it possible to simulate ddRAD-Seq *in silico*, allowing prediction of the numbers, sizes, and genome positions of digested fragments. Based on *in silico* analysis, the optimal restriction enzymes for ddRAD-Seq analyses are chosen.^10^ However, few reports have evaluated the *in silico* predictions by comparative experiments using several combinations of restriction enzymes and multiple samples with different SNP density. Moreover, it remains unclear what fraction of the SNPs in the whole genome can be detected by ddRAD-Seq. In this study, we performed *in silico* simulation of ddRAD-Seq analysis in tomato (*Solanum lycopersicum*) and validated the predictions by empirical ddRAD-Seq data using an optimized protocol. We selected tomato for this demonstration because of the richness of available genome information^14^ and the diversity of available tomato lines.^15^ In addition, we investigated the numbers of SNPs detected by ddRAD-Seq in six inbred tomato lines with different densities of genome-wide SNPs. To evaluate the quality of the SNPs, we performed linkage analyses of the SNPs identified in an F_2_ mapping population and constructed genetic linkage maps. Finally, we proposed an analytical workflow for the ddRAD-Seq procedure including a pipeline for data processing.

## 2. Materials and methods

### 2.1. Processing data for whole-genome sequence of tomato

Two tomato lines, Micro-Tom and Regina, were used as controls for empirical and *in silico* ddRAD-Seq and establishment of computational data processing pipelines. Published WGS data for Micro-Tom (accession number of DRX020765: Illumina data)^16^ and Regina (accession numbers of DRX011585 and DRX011586: SOLiD data)^15^ were used to generate a genome-wide SNP dataset. The WGS reads of the two lines were treated to remove low-quality reads and to trim adapters as described below (Computational processing for data from empirical ddRAD-Seq analysis), and mapped onto the tomato (cultivar Heinz 1706) reference genome sequences, version SL2.50, with Bowtie2 (version 2.2.3; parameters: -I 100 -X 500)^17^ and Bowtie (version 1.0; parameters: -l 15 -e 1,000),^18^ respectively. Subsequent SNP calling was also performed as below (Computational processing for data from empirical ddRAD-Seq analysis).

The genome sequence of tomato (SL2.50; https://www.sgn.cornell.edu) as well as those of *Arabidopsis thaliana* (TAIR10; https://www.arabidopsis.org), *Lotus japonicus* (build 3.0; http://www.kazusa.or.jp/lotus), and *Oryza sativa* (Os-Nipponbare-Reference-IRGSP-1.0; http://rapdb.dna.affrc.go.jp) were *in silico* treated with five restriction enzymes, e.g. *Eco*RI (recognition at site G↓AATTC), *Hin*dIII (A↓AGCTT), *Msp*I (C↓CGG), *Pst*I (CTGCA↓G), and *Sal*I (G↓TCGAC): the genome sequence was digested into restriction fragments at the points of the recognition sites of the enzymes, and information on sizes of each fragment was retained.

### 2.2. Plant materials

Six inbred tomato lines (Ailsa Craig, Micro-Tom, M82, Moneymaker, Regina, and San Marzano) were used for ddRAD-Seq analysis. All lines except for Regina were obtained from the National BioResource Project through the University of Tsukuba, Japan (accession numbers: Micro-Tom, TOMJPF00001; Moneymaker, TOMJPF00002; Ailsa Craig, TOMJPF00004; and M82, TOMJPF00005) and the Tomato Genetic Resource Center, University of California, Davis, USA (San Marzano, LA3008). Regina was commercially available from Sakata Seed Corporation (Yokohama, Japan). An F_2_ mapping population RMF2, consisting of 96 lines, was derived from a cross between Micro-Tom and Regina. Genomic DNAs were isolated from leaves of each line using the DNeasy Plant Mini Kit (Qiagen, Hilden, Germany), and quantitated using a Qubit fluorometer (Life Technologies, Carlsbad, CA, USA).

### 2.3. ddRAD-Seq analysis

A total of 250 ng of genomic DNA for each line was double digested with *Sal*I and *Pst*I, *Pst*I and *Eco*RI, *Eco*RI and *Hin*dIII, or *Pst*I and *Msp*I (FastDigest restriction enzymes; Thermo Fisher Scientific, Waltham, MA, USA); ligated to adapters (Table 1) using the LigaFast Rapid DNA Ligation System (Promega, Madison, WI, USA); and purified using Agencourt AMPure XP (Beckman Coulter, Brea, CA, USA) to eliminate short (<300 bp) DNA fragments. Purified DNA was diluted with H_2_O and amplified by PCR with indexed primers (Table 1 and Supplementary Table S1). The PCR mixture (50 µl) contained 0.4 ng of DNA, 0.2 µM of each indexed primer (one pair per mixture), 1× PCR buffer for KOD –plus– Ver. 2 (Toyobo, Osaka, Japan), 160 µM dNTPs, 1 mM MgSO_4_, and 1 U DNA polymerase (KOD –plus–; Toyobo). Thermal cycling conditions were as follows: a 3 min initial denaturation at 95°C; 20 cycles of 30 s of denaturation at 94°C, 30 s of annealing at 55°C, and a 60 s extension at 72°C; and a final 3 min extension at 72°C. Amplicons were pooled and separated on a BluePippin 1.5% agarose cassette (Sage Science, Beverly, MA, USA), and fragments of 300–900 bp were purified using the QIAGEN Mini Elute Kit (Qiagen). Concentrations of the resultant libraries were measured using the KAPA Library Quantification Kit (KAPA Biosystems, Wilmington, MA, USA) on an ABI-7900HT real-time PCR system (Life Technologies). Nucleotide sequences of the libraries were determined on a MiSeq (Illumina) in paired-end, 250 bp mode.

**Table 1.** Sequences of oligonucleotides used in ddRAD-Seq

Names	Sequence (5′ – 3′)
Restriction enzyme	
*Pst*I	TCTTTCCCTACACGACGCTCTTCCGATCTGCA
	GATCGGAAGAGCGTCGTGTAGGGAAAGAGTGT
*Eco*RI	CTGGAGTTCAGACGTGTGCTCTTCCGATCT
	AATTAGATCGGAAGAGCACACGTCTGAACTCCAGTCAC
*Hin*dIII	TCTTTCCCTACACGACGCTCTTCCGATCT
	AGCTAGATCGGAAGAGCGTCGTGTAGGGAAAGAGTGT
*Sal*I	CTGGAGTTCAGACGTGTGCTCTTCCGATC
	TCGAGATCGGAAGAGCACACGTCTGAACTCCAGTCAC
*Msp*I	CTGGAGTTCAGACGTGTGCTCTTCCGATCT
	CGAGATCGGAAGAGCACACGTCTGAACTCCAGTCAC
Indexed primers for PCR^a^	
Forward primer	AATGATACGGCGACCACCGAGATCTACACXXXXXXXXACACTCTTTCCCTACACGACGCTCTTCC
Reverse primer	CAAGCAGAAGACGGCATACGAGATXXXXXXXXGTGACTGGAGTTCAGACGTGTGCTCTTC

^a^Index bases are indicated by X, which sequences are listed in Supplementary Table S1.

### 2.4. Computational processing for data from empirical ddRAD-Seq analysis

In ddRAD-Seq data analysis as well as WGS ( Processing data for whole-genome sequence of tomato), low-quality sequences were removed and adapters were trimmed using PRINSEQ (-trim_right 1 -trim_qual_right 10 -min_len 100 -derep) and fastx_clipper (-a AGATCGGAAGAGC -l 100 -M 10 -n) in FASTX-Toolkit (http://hannonlab.cshl.edu/fastx_toolkit; version 0.10.1). The filtered reads, or subsets of the reads randomly selected using seqtk (https://github.com/lh3/seqtk), were mapped onto the reference sequences of either contigs generated by assembly of the filtered reads using Newbler (version 3.0; parameters: off for extend low depth overlap; Roche, Basel, Switzerland) or the tomato genome sequence (SL2.50) using Bowtie 2 (version 2.1.0; parameters: --minins 100 --no-mixed).^17^ The resultant sequence alignment/map format (SAM) files were converted to binary sequence alignment/map format files and subjected to SNP calling using the mpileup option of SAMtools (version 0.1.19; parameters: -Duf)^19^ and the view option of BCFtools (parameters: -vcg). Lengths of genome regions covered with more than one read at least were calculated with genomeCoverage option of BEDtools (version 2.17.0; parameters: -d).^20^ Furthermore, variant call format (VCF) files were filtered with VCFtools (version 0.1.11; parameters: --minQ 10 --minDP 4 for the cultivars' data, or --minQ 10 --minDP 4 --max-missing 0.2 --remove-indels for the RMF2 data).^21^ Missing data were imputed using Beagle4.^22^ The locations of SNPs in genic and intergenic regions were predicted using SnpEff (version 4.0e; parameters: -v SL2.50, -no-downstream and -no-upstream),^23^ and those in repetitive sequences and non-repetitive were classified in accordance with the annotation by International Tomato Annotation Group (ITAG2.4_repeats.gff3 available from Sol Genomics Network; https://www.sgn.cornell.edu). Similarity searches of marker-associated sequences against the SL2.50 tomato genome sequence were carried out using BlastN with default parameters.^24^

### 2.5. Linkage analysis and construction of genetic linkage maps

Linkage analysis was carried out with the imputed SNP dataset for RMF2. The segregated data were classified into groups using the grouping module of JoinMap4^25^ with LOD scores of 3–6. The marker order and relative map distances were calculated using the regression-mapping algorithm with the following parameters: Haldane's mapping function, recombination frequency ≤0.35, and LOD score ≥2.0. The graphical maps were drawn using the MapChart program.^26^

## 3. Results

### 3.1. Establishment of a genome-wide SNP dataset

WGS data for Micro-Tom^16^ and Regina^15^ were used to generate a genome-wide SNP dataset by mapping the reads onto the tomato reference sequence, version SL2.50,^14,27^ as described in Materials and Methods. Mapping rate and fraction of aligned regions of the SL2.50 were 97.9 and 99.2%, respectively, in Micro-Tom, while those were 61.1 and 98.8%, respectively, in Regina. A total of 1,187,941 high-quality SNPs between the two lines were discovered by filtering with the following parameters (Supplementary Table S2): SNP quality, >10, and depth of coverage, ≥4. The SNP loci were unevenly distributed over the genome, with numbers ranging from 10,170 SNPs on chromosome 6 (chromosome length of 49.8 Mb in total) to 277,708 SNPs on chromosome 4 (66.5 Mb in total) (Supplementary Table S2). Only 13.5 and 39.1% of the 1,187,941 SNPs were found on genic regions and non-repeat sequences (Supplementary Fig. S1 and Table S2), respectively, both of which are biologically important sequences in the genome (see Distribution of SNPs in genic/intergenic regions and repeat/non-repeat sequences for details).

### 3.2. *In silico* restriction digestion to determine optimal restriction enzymes

**Figure 1.**
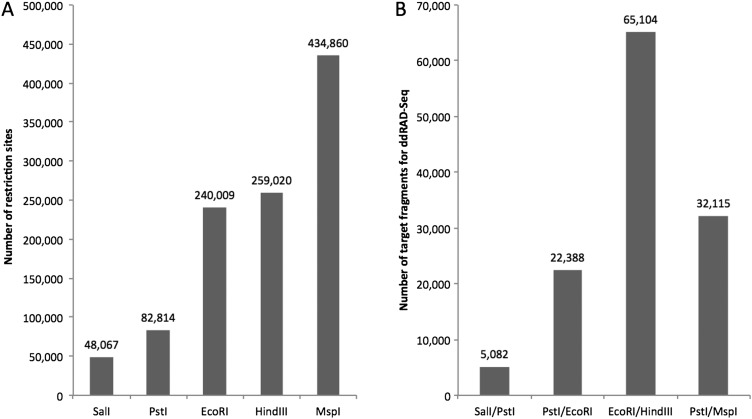
Numbers of restriction sites and restriction fragments in the tomato genome (SL2.50). Bars indicate the numbers of restriction sites (A) and 300–900 bp restriction fragments (B) predicted from the SL2.50 tomato genome sequence by *in silico* analysis.

To identify the optimal restriction enzymes for experimental ddRAD-Seq analysis, we performed *in silico* restriction digestion. For this analysis, we selected five enzymes (*Sal*I, *Pst*I, *Eco*RI, *Hin*dIII, and *Msp*I) with different frequencies of recognition sites in the tomato genome, low in *Sal*I and *Pst*I, middle in *Eco*RI and *Hin*dIII, and high in *Msp*I (Fig. 1A). Four combinations of the enzymes (a combination of low and low: *Sal*I/*Pst*I; low and middle: *Pst*I/*Eco*RI; middle and middle: *Eco*RI/*Hin*dIII; and middle and high: *Pst*I/*Msp*I) were used for *in silico* digestion of the genome sequence. The numbers of fragments with 300–900 bases, our target for experimental ddRAD-Seq experiment, covered the entire tomato genome evenly (Supplementary Fig. S2), but varied from 5,082 for *Sal*I/*Pst*I to 65,104 for *Eco*RI/*Hin*dIII (Fig. 1B and Supplementary Fig. S2 and Table S2). The distributions of SNPs on each chromosome corresponded to those obtained from WGS data, although the total numbers of the SNPs decreased drastically, to only 3,553 (0.3%) for *Sal*I/*Pst*I and 47,768 (4.0%) for *Eco*RI/*Hin*dIII (Supplementary Fig. S1 and Table S2). While proportions of SNPs on genic regions to the detected SNPs were ranging from 16.3% (*Eco*RI/*Hin*dIII) to 29.0% (*Pst*I/*Eco*RI), those of SNPs in non-repeat sequences to whole genome were from 26.7% (*Sal*I/*Pst*I) to 45.0% (*Pst*I/*Eco*RI) (Supplementary Fig. S1 and Table S2). The *in silico* analysis was applied to other plant species, e.g. *A. thaliana*, *L. japonicus*, and *O. sativa*. The result indicated that the tendency was similar to those of *A. thaliana* and *L. japonicus* except for *O. sativa* in which number of *Pst*I/*Msp*I fragments were predominant (Supplementary Fig. S3).

### 3.3. Establishment of data processing pipeline for ddRAD-Seq

A data processing pipeline for SNP discovery was established using actual MiSeq reads of Micro-Tom and Regina ddRAD-Seq libraries generated using the *Pst*I/*Msp*I combination (PM libraries). Briefly, sequence reads were processed by removing low-quality reads and trimming adapters, and then mapped onto the reference sequence to detect SNP candidates (see Materials and Methods for details). When 1.9 and 2.2 M paired-reads for Micro-Tom and Regina, respectively, were analysed using this pipeline, 1.2 and 1.4 M high-quality reads were obtained, and 83,011 SNP candidates, including 20,689 homozygous and 62,322 loci with genotypes called as ‘heterozygous’, were detected prior to filtering. Because the two lines are inbred, the ‘heterozygous’ SNPs were excluded because they were likely to reflect sequencing or alignment errors. Of the 20,689 homozygous SNPs, 19,969 SNPs with quality values >10 were selected as high-confidence SNP loci. Out of them, 15,746 SNPs (78.9%) were identical to those from WGS data, whereas the remaining 4,223 SNPs (22.1%) were not found due to sites of insufficient read coverage in the WGS data.

### 3.4. Experimental validation of SNP candidates detected by *in silico* analysis

**Figure 2.**
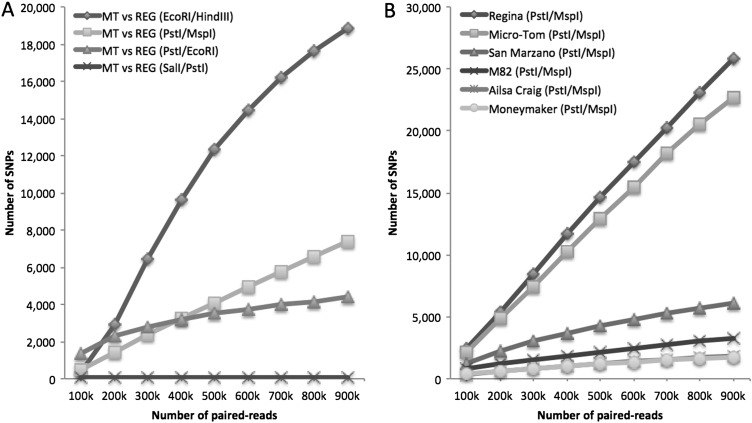
Number of SNPs detected from empirical ddRAD-Seq analysis. Line chart indicates numbers of SNPs between Micro-Tom and Regina with four combinations of restriction enzymes (A) and SNPs of six cultivars with respect to SL2.50 using the *Pst*I/*Msp*I combination (B).

To validate the accuracy of the *in silico* predictions that the numbers of SNPs detected in ddRAD-Seq would be depending on choice of restriction enzymes, additional MiSeq reads were obtained from Micro-Tom and Regina ddRAD-Seq libraries generated using three more restriction enzyme combinations, *Sal*I/*Pst*I (SP), *Pst*I/*Eco*RI (PE), and *Eco*RI/*Hin*dIII (EH) as well as PM as above. After removal of low-quality sequences and trimming of adapters, a subset of 100k to 900k high-quality paired-end reads were generated for all four libraries. Each subset was mapped onto the reference genome sequence, SL2.50, and high-quality SNP candidates were selected by filtering using the criteria described above. As expected, the number of SNPs in each dataset increased as the number of reads increased (Fig. 2A). However, this tendency differed considerably among the enzyme combinations. The number of SNPs of PM increased linearly up to ∼8,000 when 900k reads were used, whereas that of EH gradually reached ∼20,000. In contrast, despite their higher numbers of reads, the PE and SP libraries had far fewer SNPs: 4,000 for PE and <100 for SP. As in the *in silico* prediction, the EH and PM libraries gave much more SNPs than the PE and SP.

### 3.5. Distribution of SNPs in genic/intergenic regions and repeat/non-repeat sequences

**Figure 3.**
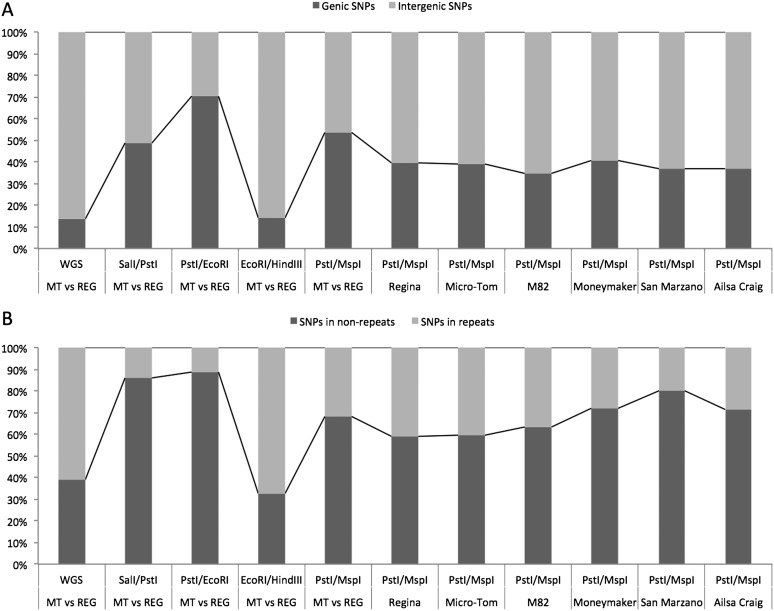
Proportions of SNPs detected from empirical ddRAD-Seq analysis. SNPs from empirical ddRAD-Seq libraries are distributed in genic and intergenic regions (A) and repeat and non-repeat sequences (B). Proportions of SNPs between Micro-Tom and Regina (MT vs REG) detected from WGS data is shown as a control.

Since SNPs in genes and non-repetitive sequences are biologically meaningful in comparison with those in intergenic and repetitive regions. Not only proportions of genic and intergenic SNPs detected in the empirical ddRAD-Seq but also those of unique and repeat sequences in the tomato genome were investigated. The result indicated remarkable differences of the proportions among the restriction enzyme combinations (Fig. 3A). In the PE libraries, 70.1% of SNPs were derived from genic regions. This rate is much higher than those from the *in silico* prediction, which suggested that 29.0% of SNPs occurred in genic regions (Supplementary Fig. S1). Furthermore, the PM and SP libraries were enriched for genic SNPs. In contrast, the EH library had SNP frequencies comparable with those obtained from the prediction. The proportions of the SNPs in the repeat/unique sequences were also markedly different among the libraries (Fig. 3B and Supplementary Fig. S1): SNPs from the SP, PE, and PM libraries were enriched in the unique sequences in comparison with the prediction, while the proportion of the EH library was comparable with the prediction. We concluded that the PM and PE libraries had advantages to detect SNPs in gene regions and non-repetitive sequences in the tomato genome.

### 3.6. ddRAD-Seq in genetically diverse tomato lines

Micro-Tom and Regina show larger genetic distances to Heinz 1706 in comparison with the other cultivated tomato lines.^28^ To assess the numbers of SNPs in genetically diverse samples, the six lines, i.e. Ailsa Craig, M82, Moneymaker, and San Marzano as well as Micro-Tom and Regina, were further analysed with ddRAD-Seq. The *Pst*I/*Msp*I combination was employed in accordance with the results of the validation test, expecting to gain as many SNPs in genes and unique sequences of the tomato genome as possible. The high-quality sequence data from the six PM libraries were divided into subsets of 100k–900k paired-end reads and mapped onto the reference genome. As expected, the numbers of SNPs with respect to Heinz 1706 (SL2.50) detected by experimental ddRAD-Seq in Regina and Micro-Tom increased linearly up to ∼25,000, whereas those in the other four cultivars reached 5,000 or less (Fig. 2B), indicating that SNP density in the genome influenced the detectability of SNPs by ddRAD-Seq. A graphical genotypes based on the result from the ddRAD-Seq with 900k paired-end reads indicated that the distribution of the SNPs was highly biased on the genome as reported in our previous study (Supplementary Fig. S4).^14^ The SNP densities relative to SL2.40, a previous version of the tomato genome sequence with the same base compositions to the SL2.50,^27^ were estimated to be one SNP per 651 bp in Regina,^15^ 803 bp in Micro-Tom,^16^ 1,011 bp in M82,^15^ 3,105 bp in Moneymaker,^29^ 4,347 bp in San Marzano,^30^ and 8,387 bp in Ailsa Craig.^15^ The percentages of SNPs detected by ddRAD-Seq analysis per genome-wide SNPs by the WGS were almost even in these six lines: 2.0% genome-wide SNPs on average, ranging from 0.5% in M82 to 3.6% in San Marzano, and proportion of the SNPs in gene regions and repeat sequences from the six lines were similar to those from a combination of Micro-Tom and Regina (Fig. 3A and B).

### 3.7. ddRAD-Seq in an F_2_ mapping population to construct genetic maps

Accuracy of SNP genotypes called from the ddRAD-Seq pipeline was validated by construction of genetic linkage maps. Because miss-called SNPs would be rejected from the maps, mapping rate of SNPs is an indicator of the accuracy.

**Figure 4.**
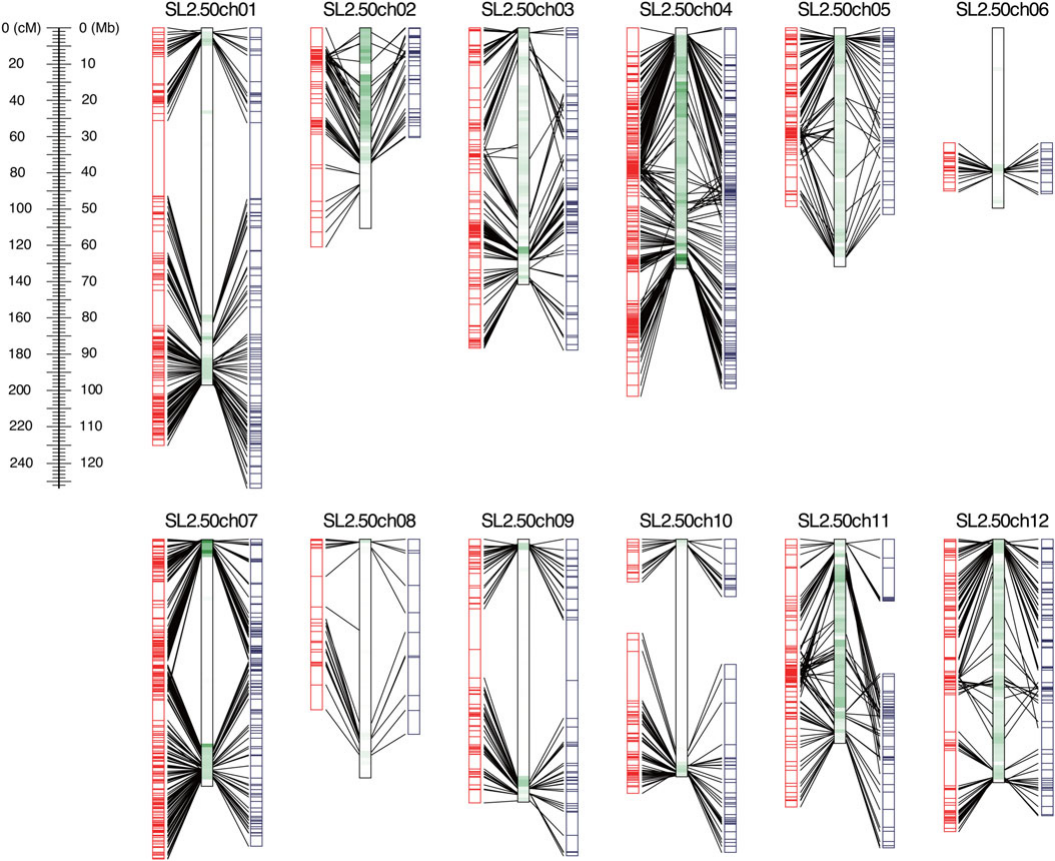
Genetic linkage maps of RMF2, an F_2_ population derived from a cross between Micro-Tom and Regina. Bars on the left and right sides indicate linkage group maps based on SNP loci detected in the tomato reference genome (red lines) and a *de novo* assembly of ddRAD-Seq data (blue lines). Bars between the two maps indicate the physical map of the tomato genome. The density of SNPs detected using WGS data for the two cultivars is indicated by the darkness of green lines. Loci that are identical between the genetic and physical maps are connected by lines.

For the same reason as above to obtain as many SNPs as possible from gene and non-repetitive regions, the *Pst*I/*Msp*I was selected as the optimal enzyme combination for library construction for the F_2_ mapping population (*n* = 96), RMF2, derived from a cross between Micro-Tom and Regina. Ninety-six libraries of RMF2 with index tags distinguishing each line (Table 1 and Supplementary Table S1) were pooled and sequenced on an Illumina MiSeq, yielding an average of 268k paired-end reads (=134 Mb, 0.14× genome coverage) per line. After removal of low-quality sequences and trimming of adapters, 226k high-quality reads on average in each line were mapped onto SL2.50 along with the reads from the parental lines. Mapping rates of Micro-Tom and Regina were 94.3 and 92.9%, respectively, while that in the F_2_ population was 91.0% on average. Of 155,992 SNP candidates between the parental lines, 60,512 loci with quality of ≤10 and depth of <4 were eliminated; furthermore, 89,241 ‘heterozygous’ SNPs, probably resulting from sequencing and/or alignment errors as noted above, were also removed. Ultimately, 6,239 loci were selected. By allowing 20% missing data for each SNP locus across the 96 F_2_ lines, 1,845 positions (depth of coverage of 13.5 on average) of the 6,239 loci were selected as high-confidence segregating SNPs in the F_2_ population. Prior to linkage analysis, the missing genotypes were imputed in accordance with genotype data from the parental lines. Subsequently, 528 genetic loci similar to others were eliminated. Of the remaining 1,317 non-redundant SNP loci, 1,297 (98.5%) were classified into 13 groups, each of which corresponded to one tomato chromosome (with the exception of chromosome 10, which was represented by two groups). Linkage analysis generated a genetic map consisting of 13 linkage groups, with 1,257 loci (95.4%) covering a total of 1,693.2 cM (Table 2 and Fig. 4). The distributions of mapped loci were biased both inter- and intra-chromosomally, reflecting the biases in genome-wide SNP distributions. The order of the mapped loci were consistent with their physical positions in SL2.50 (Fig. 4).

**Table 2.** Number of mapped loci and length of genetic linkage maps

Linkage group	Reference-based map		*De novo* map	
	#Mapped loci	Map length (cM)	#Mapped loci	Map length (cM)
1	151	230.1	86	253.6
2	58	120.9	32	60.6
3	126	176.7	66	177.8
4	240	203.3	139	199.0
5	85	98.7	38	103.0
6	25	26.6	13	28.1
7	212	176.4	99	169.4
8	25	94.1	11	107.6
9	70	145.5	44	174.6
10	68^a^	111.8^a^	43^a^	135.4^a^
11	93	147.7	50^a^	130.2^a^
12	104	161.3	65	152.5
Total	1,257	1,693.2	686	1,691.8

^a^These numbers reflect the total values of divided linkage groups.

Next, we investigated the accuracy of SNP calling without a reference genome sequence. The experimental ddRAD-Seq reads of the parental lines were assembled *de novo* into 44,764 contigs with a total length of 12,443,360 bases, and the high-quality ddRAD-Seq reads for the parents and the 96 F_2_ lines were subsequently aligned onto the contigs with mapping rates of 65.0% in Micro-Tom, 62.8% in Regina, and 59.0% in the F_2_ population. The genome positions of the marker loci on the tomato genome were determined by sequence similarity searches against the SL2.50 sequences. Using the same filtering process described above, a total of 1,017 high-confidence SNPs were selected between the parents, and 781 were identified as non-redundant SNP loci in RMF2. Linkage analysis of the 781 SNPs generated a genetic map comprising 14 linkage groups (Table 2 and Fig. 4), each of which corresponded to one tomato chromosome (except for chromosomes 10 and 11, which were represented by two groups apiece). The resultant map consisted of 686 SNP loci (87.8%) covering a total of 1,691.8 cM, and the order of the loci were consistent with their physical positions in the reference genome (Fig. 4). As for the SNPs identified on SL2.50, the distributions of mapped loci were highly biased both between chromosomes and within individual chromosomes. The two mapping studies indicated that the accuracy of SNPs from our ddRAD-Seq pipeline was ∼90% or more.

## 4. Discussion

**Figure 5.**
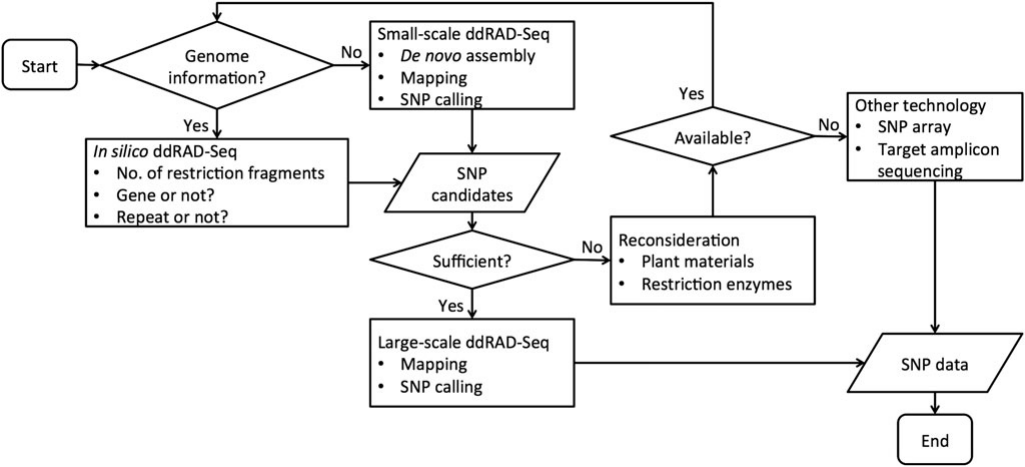
The ddRAD-Seq analytical workflow based on empirical and *in silico* optimization.

We propose an analytic workflow for the ddRAD-Seq procedure (Fig. 5). During the establishment experimental and computational data processing pipelines, we found that the prediction of SNP detectability in ddRAD-Seq facilitated optimization of experimental conditions, e.g. choices of enzymes and the density of SNPs in the genome. Although all libraries contained the same amount of sequence data, the numbers of SNPs detected by experimental ddRAD-Seq varied depending on both the combination of restriction enzymes and the density of SNPs in the genome (Fig. 2A and B). *In silico* prediction should be also useful for optimizing experimental conditions in other plant species for which reference genome sequences and sequencing data are available. On the other hand, in plant species for which less genomic information has accumulated, small-scale pilot experiments with several combinations of restriction enzymes should be performed to determine the optimal enzymes for ddRAD-Seq experiment.

Gene-associated SNPs located on non-repetitive sequences would be biologically meaningful, being beneficial for functional genomics, molecular genetics, and marker-assisted selection in breeding. Interestingly, the rate of gene SNPs detected by the empirical ddRAD-Seq was higher than the predicted rate when *Pst*I was employed for library construction, e.g. PE, PM, and SP libraries (Fig. 3A, B and Supplementary Fig. S1). Therefore, this point as well as the number of SNPs should be considered to select optimum restriction enzymes. It seems likely that the strong enrichment of euchromatic genes in the libraries is correlated to the methylation sensitivity of restriction enzymes.^5^ Whole-genome bisulphite sequencing analysis would be helpful to verify this hypothesis.

Genome complexity, i.e. ploidy and zygosity, is another important factor that influences the choice of restriction enzymes. For inbred lines and haploids without any heterozygous loci, SNP loci can in principle be correctly genotyped with coverage of at least one high-quality read. In such cases, to obtain as many SNPs as possible, a combination of enzymes should be selected that yields SNP numbers that increase linearly with the number of sequence reads (EH library in Fig. 2A). In contrast, plants with highly heterozygous genomes, e.g. hybrid and polyploid lines, require deep read coverage for accurate SNP detection. Therefore, to distinguish homo- and heterozygous genotypes (or, for polyploids, homologous and homoeologous genotypes), an enzyme combination should be selected that yields a gradually increasing number of SNPs (PE library in Fig. 2A).

Reference sequences are essential for SNP detection, but they remain unavailable for many plant species. In the absence of a reference sequence, *de novo* assembly of actual ddRAD-Seq reads should be used as a reference. To simulate this situation, we performed *de novo* assembly of the ddRAD-Seq reads generated in this study. The numbers of high-quality SNP loci, non-redundant segregated data, and SNPs located on genetic maps based on the *de novo* assemblies were ∼50% of those based on the SL2.50 reference (Table 2). However, the total lengths of the resultant genetic maps were almost identical, indicating that both genetic maps were saturated. These results indicate that *de novo* assembly of the ddRAD-Seq reads is sufficient to establish saturated genetic maps. Alternatively, considering recent advances in NGS technologies, whole-genome sequence data from close relatives of a target species might be available,^13^ and it is generally also possible to generate WGS of the target species itself.

The numbers of SNPs detected by ddRAD-Seq varied depending on SNP density in the genome (Fig. 2B). In other words, SNP density is a key factor influencing SNP detectability by ddRAD-Seq. Unfortunately, a strong bias in distribution of SNPs over the genome was observed between Micro-Tom and Regina (Fig. 4 and Table 2), the resultant genetic map with large gaps failed to cover the entire genome. Therefore, either the *in silico* ddRAD-Seq analysis or small-scale experiments with several combinations of restriction enzymes are recommended to predict SNP availability from actual large-scale ddRAD-Seq analysis before generating mapping populations. However, if this is impossible, increasing the variety of sequencing libraries is another possible way to increase the numbers of SNPs. For instance, although 0.3% (SP) to 4.6% (EH) of SNPs in the genome were theoretically detectable using a single sequencing library, this fraction reached a maximum of 7.6% when four libraries (SP, PE, EH, and PM) were analysed simultaneously. For plants with ultra-low SNP density in the genomes from which few SNPs are expected, alternatively, the SNP chip technologies and/or target capture or target amplicon sequencing technology,^31^ which tags SNPs regardless of their distances from restriction sites, might be useful; however, this approach would be more costly than ddRAD-Seq. Therefore, prediction of the expected number of SNPs based on SNP density throughout the genome would be helpful to maximize the efficiency of ddRAD-Seq analysis.

In conclusion, the ddRAD-Seq technology has the potential to simultaneously genotype SNPs throughout the genome in multiple samples.^5,6,8,10^ The ddRAD-Seq analytical workflow and the pipeline for the data processing developed in this study (Fig. 5), including the empirical and *in silico* optimization processes, could be used to advance genetics, genomics, and molecular breeding in both model and non-model plant species, including crops.

## 5. Availability

All sequence data obtained in this study are available from the DDBJ Sequence Read Archive under accession number DRA003569 and Kazusa Tomato Genomics DataBase (KaTomicsDB: http://www.kazusa.or.jp/tomato).^32^

## Supplementary Data

Supplementary Data are available at www.dnaresearch.oxfordjournals.org.

## Funding

Funding to pay the Open Access publication charges for this article was provided by the Kazusa DNA Research Institute.

## Supplementary Material

Supplementary Data
