# Supplementary material for: Analytical workflow of double-digest restriction site-associated DNA sequencing based on empirical and in silico optimization in tomato
Source: DNA Res. 2016 Feb 29;23(2):145–53. doi: 10.1093/dnares/dsw004 (PMC4833422; doi:10.1093/dnares/dsw004)
Supplement: Supplementary Data [file supp_23_2_145__index.html]

Analytical workflow of double-digest restriction site-associated DNA sequencing based on empirical and in silico optimization in tomato — Analytical workflow of double-digest restriction site-associated DNA sequencing based on empirical and in silico optimization in tomato — Supplementary Data 

# Analytical workflow of double-digest restriction site-associated DNA sequencing based on empirical and *in silico* optimization in tomato

## Supplementary Data

Supplementary Data

- Supplementary Data - Pdf file
- Supplementary Figure 1 - pdf file
- Supplementary Figure 2 - pdf file
- Supplementary Figure 3 - pdf file
- Supplementary Figure 4 - pdf file
- Supplementary Table 1 - xlsx file
- Supplementary Table 2 - xlsx file
