# Supplementary material for: Analytical workflow of double-digest restriction site-associated DNA sequencing based on empirical and in silico optimization in tomato
Source: DNA Res. 2016 Feb 29;23(2):145–53. doi: 10.1093/dnares/dsw004 (PMC4833422; doi:10.1093/dnares/dsw004)
Supplement: Supplementary Data [file supp_dsw004_dsw004supp.pdf]

DNA Research doi: 10.1093/dnares/dsw004

Analytical workflow of double-digest restriction site-associated DNA sequencing based on empirical and *in silico* optimization in tomato

Kenta Shirasawa, Hideki Hirakawa, and Sachiko Isobe

Supporting information

Supplementary Fig. S1 Proportions of SNPs detected from *in silico* ddRAD-Seq analysis.
SNPs from *in silico* ddRAD-Seq are distributed in genic and intergenic regions (A) and repeat and non-repeat sequences (B). Proportions of SNPs between Micro-Tom and Regina (MT vs REG) detected from WGS data, which are also indicated in Fig. 3, is shown as a control.

Supplementary Fig. S2 Genome distributions of fragments and SNPs of ddRAD-Seq libraries computationally predicted from the tomato genome sequence data.

Supplementary Fig. S3 Numbers of restriction fragments in the genomes of *S. lycopersicum* (SL2.50), *A. thaliana* (TAIR10), *L. japonicus* (build 3.0), and *O. sativa* (Os-Nipponbare-Reference-IRGSP-1.0).
Bars indicate the numbers of 300–900 bp restriction fragments predicted from the genome sequences by *in silico* analysis. The data for tomato are also indicated in Fig. 1B.

Supplementary Fig. S4 Graphical genotypes of the six tomato lines tested.
Bars indicate the tomato chromosomes. Numbers on the top of the chromosomes are chromosome numbers and chromosomes number zero is unassinged contig to the tomato chromosomes. Lines on the chromosomes show SNP loci. Red and blue represent alleles of the reference genome of Heinz 1706 and those of alternative lines, respectively.
