# Supplementary figures and images for: Analytical workflow of double-digest restriction site-associated DNA sequencing based on empirical and in silico optimization in tomato
Source: DNA Res. 2016 Feb 29;23(2):145–53. doi: 10.1093/dnares/dsw004 (PMC4833422; doi:10.1093/dnares/dsw004)

A

Genic SNPs Intergenic SNPs

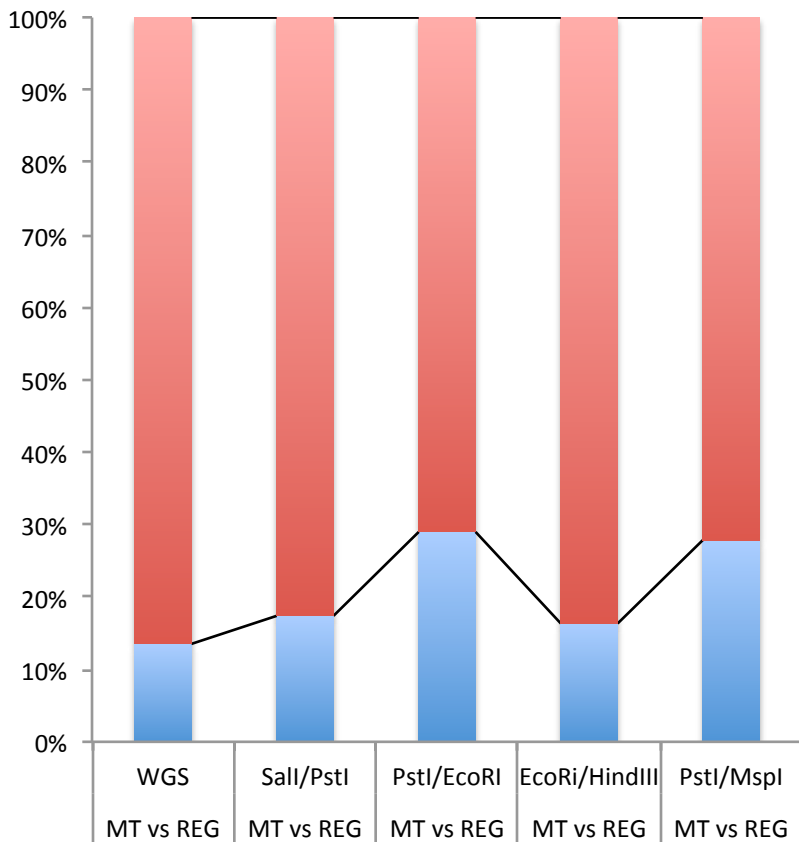

B

SNPs in non-repeats SNPs in repeats

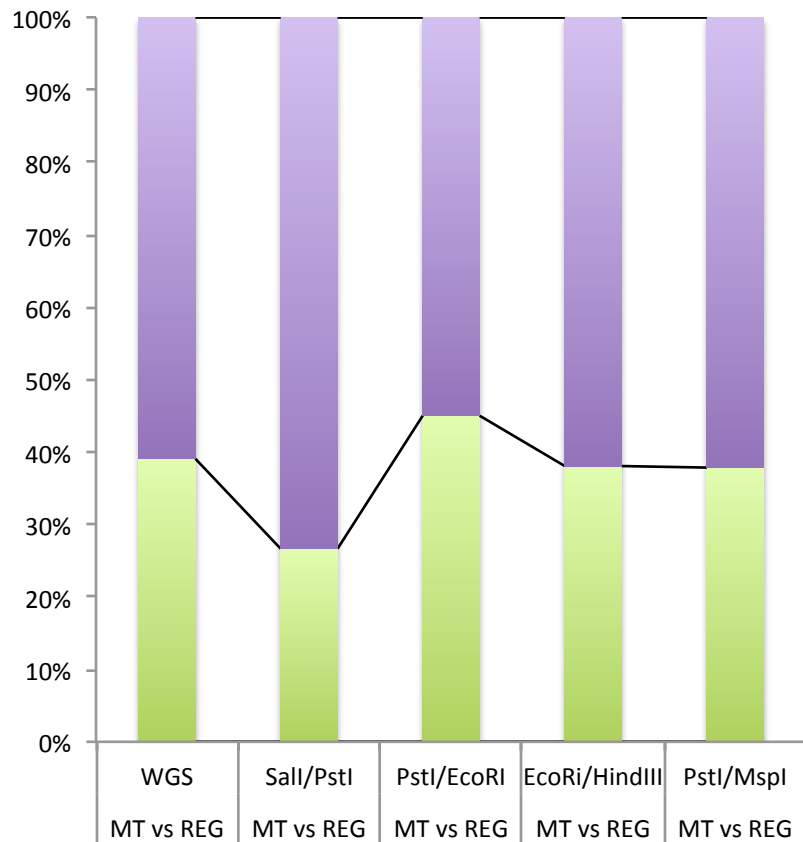

Supplement: Supplementary Data [file supp_dsw004_dsw004supp_fig1.pdf]

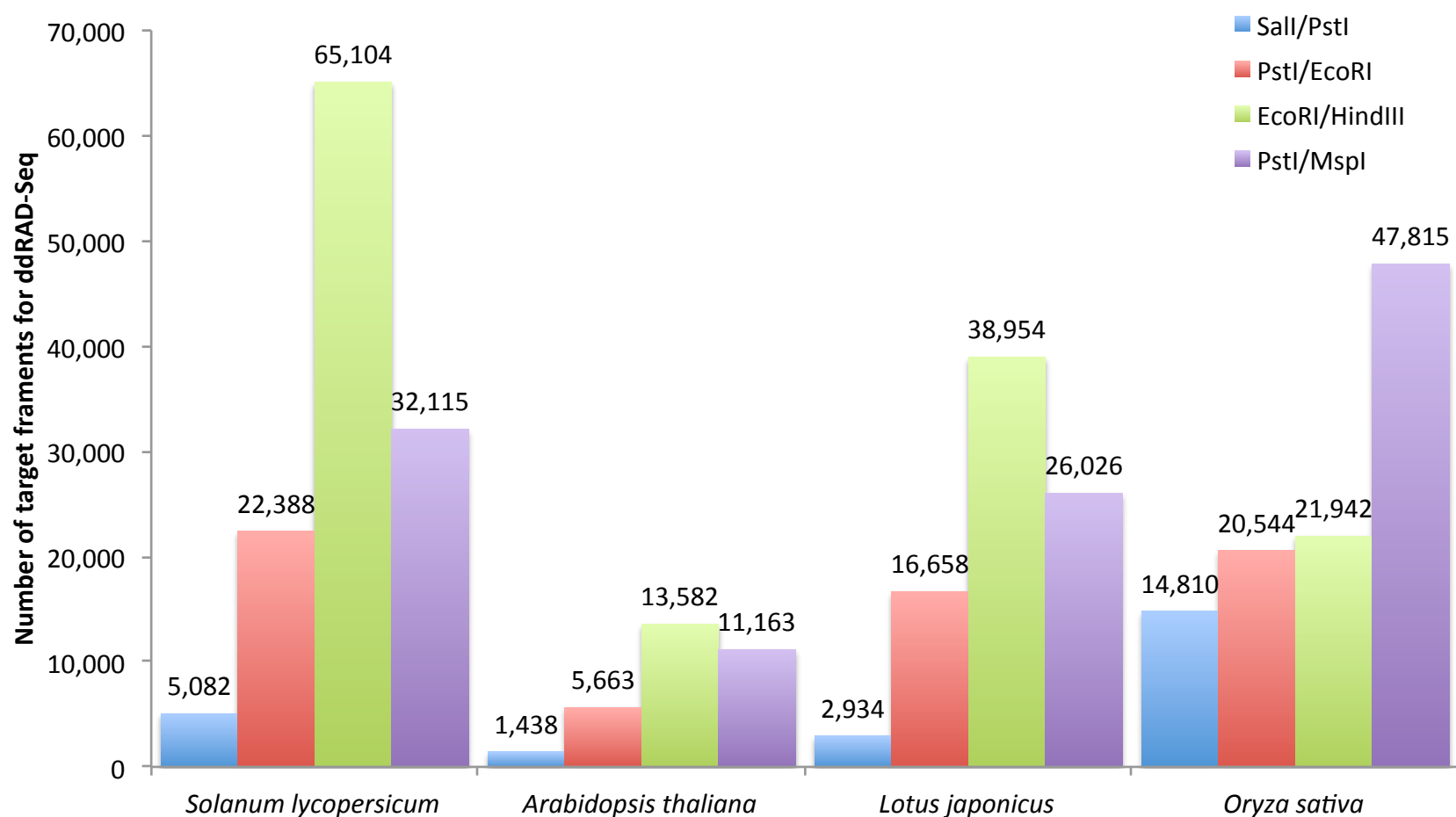

Supplement: Supplementary Data [file supp_dsw004_dsw004supp_fig3.pdf]

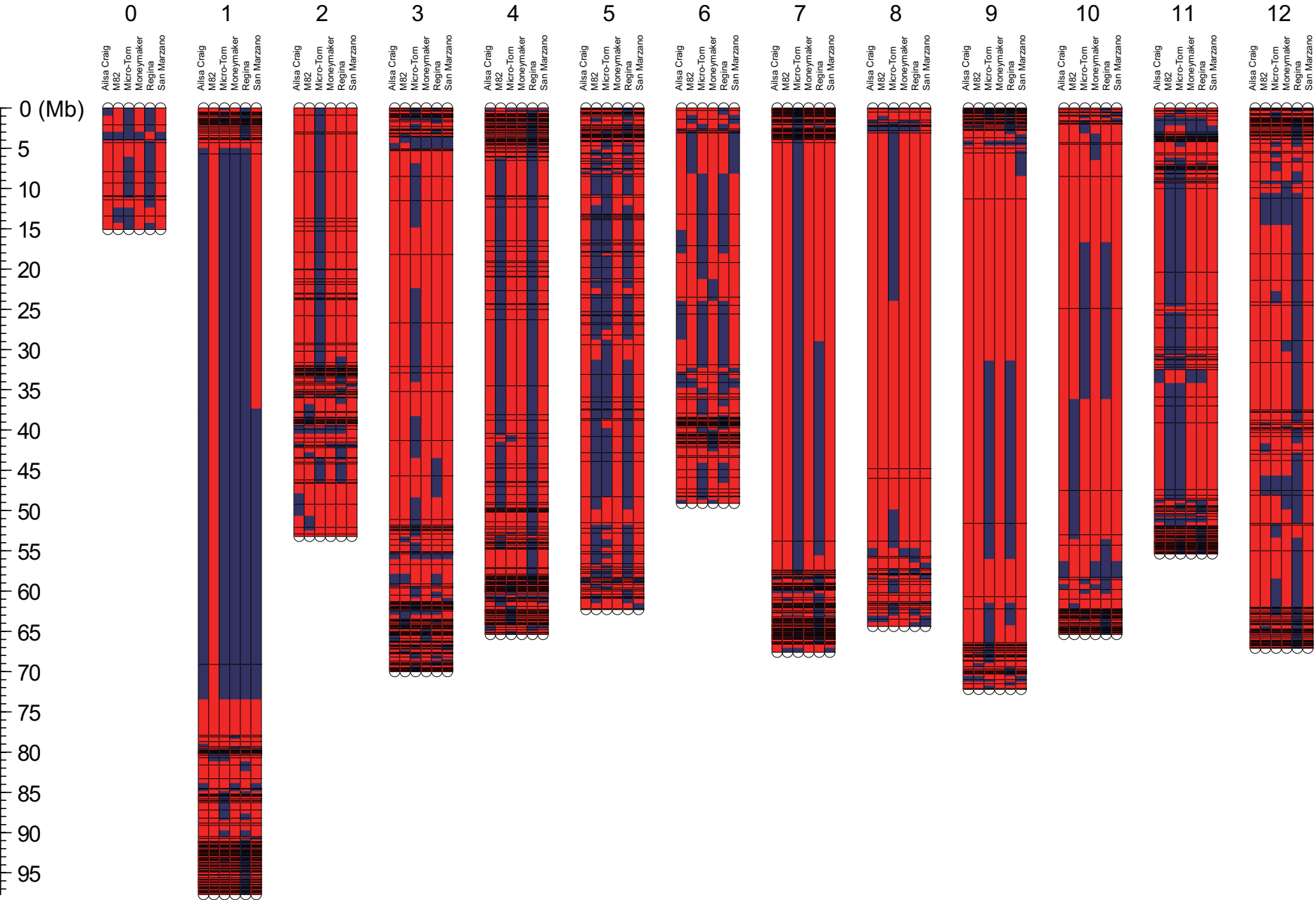

Supplement: Supplementary Data [file supp_dsw004_dsw004supp_fig4.pdf]
