# Supplementary material for: Analytical workflow of double-digest restriction site-associated DNA sequencing based on empirical and in silico optimization in tomato
Source: DNA Res. 2016 Feb 29;23(2):145–53. doi: 10.1093/dnares/dsw004 (PMC4833422; doi:10.1093/dnares/dsw004)
Supplement: Supplementary Data [file supp_dsw004_dsw004supp_fig2.pdf]

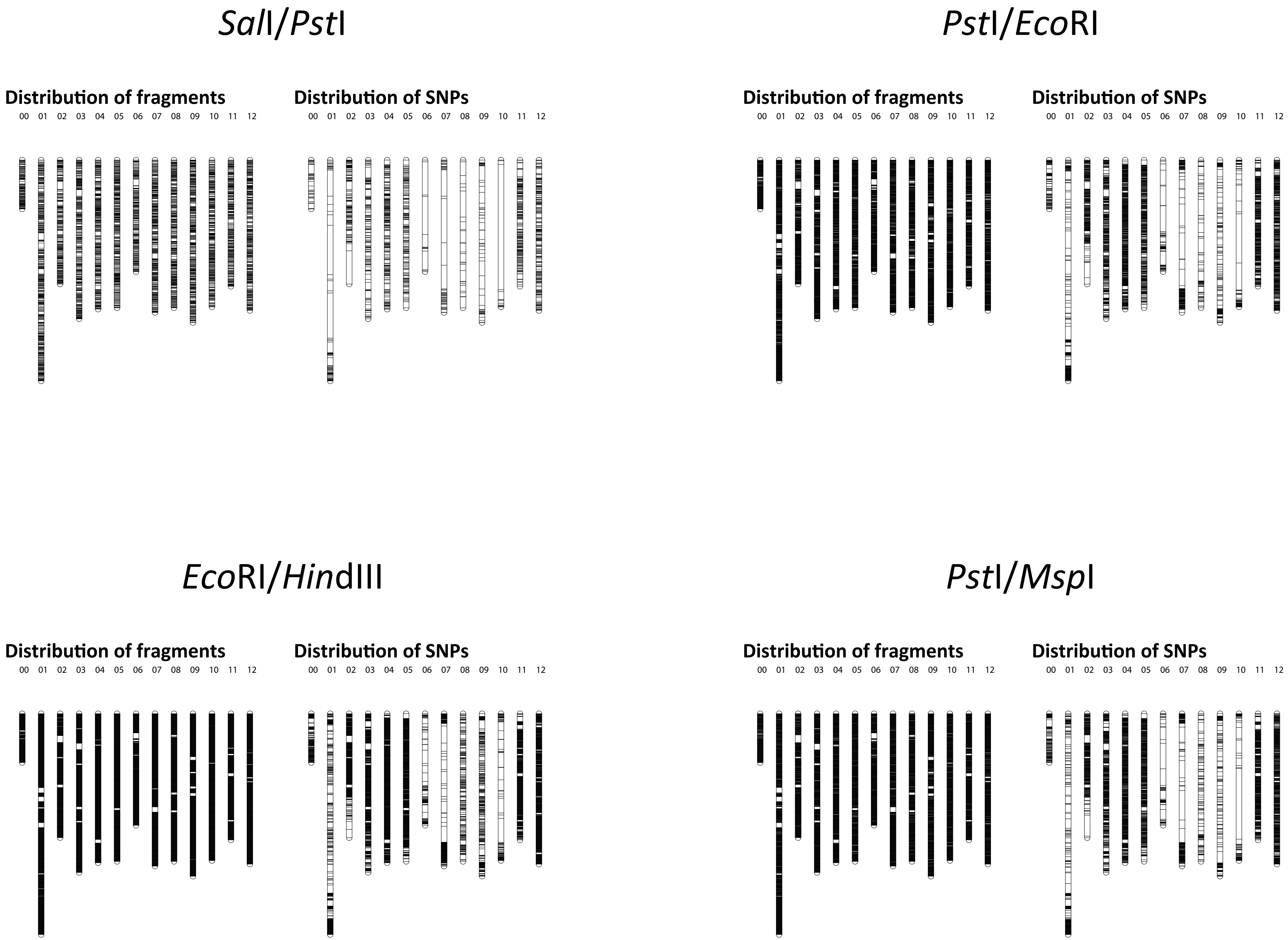

*Sal*I/*Pst*I
Distribution of fragments
Distribution of SNPs
00 01 02 03 04 05 06 07 08 09 10 11 12
*Pst*I/*Eco*RI
Distribution of fragments
Distribution of SNPs
00 01 02 03 04 05 06 07 08 09 10 11 12
*Eco*RI/*Hin*dIII
Distribution of fragments
Distribution of SNPs
00 01 02 03 04 05 06 07 08 09 10 11 12
*Pst*I/*Msp*I
Distribution of fragments
Distribution of SNPs
00 01 02 03 04 05 06 07 08 09 10 11 12
